# How much training is enough? Evaluating clinician self-reported family violence response skills following a 3-year transformational change project in a major trauma hospital

**DOI:** 10.1177/17455057241286552

**Published:** 2024-10-18

**Authors:** Caroline A Fisher, Catherine Rushan, Toni D Withiel

**Affiliations:** 1 Allied Health – Psychology, Royal Melbourne Hospital, Melbourne, VIC, Australia; 2 Allied Health – Family Safety Team, Royal Melbourne Hospital, Melbourne, VIC, Australia; 3 School of Psychology and Public Health, La Trobe University, Bundoora, VIC, Australia; 4 Neuropsychology Service, The Melbourne Clinic, Richmond, VIC, Australia

**Keywords:** domestic violence, education, family violence, hospital, training

## Abstract

**Background:**

Rates of family violence are high in many societies, with disproportionate impacts on women and children. Healthcare services have an important gateway role for victim-survivors requiring assistance. There is limited evidence regarding how much training is required for hospital clinicians to be adequately prepared to work effectively with clients experiencing family violence.

**Objectives:**

This study aimed to investigate the impact of different levels of training in family violence, on the knowledge and confidence of hospital clinicians.

**Design:**

A cross-sectional, online, survey of hospital clinicians in a major trauma hospital was conducted. The study evaluated the impact of level of family violence training (no training, some training, clinical champions) on staff self-reported family violence knowledge and confidence levels.

**Methods:**

The Assisting Patient/Clients Experiencing Family Violence: Royal Melbourne Hospital Clinician Survey tool was utilised, and open for clinicians to complete, anonymously over a 6-week period.

**Results:**

In total, 526 clinical staff participated across a range of profession groups (Allied Health, 47%; Nursing 40%; Medical 13%). Staff with some training (mean training hours 3.25, SD 5.23) rated their knowledge and confidence levels at least two-thirds higher than those with no training. Those trained as clinical champions (mean training hours 14.60, SD 9.14) rated their knowledge and confidence at least 50% higher than staff with some training. An even more pronounced elevation across training levels was seen with specific family violence clinical skills – identifying the signs of family violence, knowing how to screen patients and providing an appropriate response to disclosures.

**Conclusions:**

Training in family violence clinical response significantly increased self-reported knowledge and confidence levels of hospital staff, with the extra time and resourcing required to train clinical champions, showing clear benefits. The provision of evidence-based and well-resourced family violence education for healthcare professionals is required to drive clinical practice improvements for victim-survivors.

## Plain language summary

Training requirements for healthcare workers helping patients with family violence issues.

Rates of family violence are high in many communities. Women and children are most affected by this violence. Healthcare workers have an important role to play in helping people with family violence issues. There is little information about how much training healthcare workers need to do this effectively. This study evaluated the effect of different levels of training in family violence on the knowledge and confidence of hospital clinicians. Healthcare workers in a large trauma hospital were surveyed. 526 clinical staff responded, including Allied Health (47%), Nursing (40%), and Medical (13%) staff. Clinicians with some training rated their knowledge and confidence levels at least two-thirds higher than those with no training. Those trained as clinical champions rated their knowledge and confidence at least 50 percent higher than staff with some training. An even more pronounced difference was seen across training levels for specific family violence clinical skills. These included identifying the signs of family violence, knowing how to screen patients, and providing an appropriate response to disclosures. Overall, training in family violence clinical response significantly increased self-reported knowledge and confidence levels of clinicians, with the extra time and resourcing required to train clinical champions, showing clear benefits. Well-resourced and designed family violence education for healthcare professionals is required for appropriate care and support to be provided for victim-survivors of family violence.

## Introduction

Family violence is a public health concern affecting individuals, communities and countries around the globe. Rooted in behaviours that create an environment of fear and intimidation, family violence is broad in scope, encompassing physical, psychological, emotional, sexual and financial abuse. It includes intimate partner violence, domestic violence, coercive control, domestic abuse, child abuse and elder abuse. Family violence is predominantly perpetrated by men against women and children.^1^ However, family violence can affect people of any gender identity and sexual orientation and occurs across the lifespan.^2–5^ Family violence also includes exposure, such as when a child hears, witnesses or is impacted by the effects of abusive behaviour.^6^

Global statistics illustrate the widespread nature of this significant problem. In countries that form part of the Organisation for Economic Co-operation and Development, between 15% and 42% of women report a lifetime prevalence of physical and/or sexual violence from by an intimate partner.^7^ This has lethal consequences. In 2017, of the 87,000 women intentionally killed worldwide, 58% (i.e. 50,000) were killed by an intimate partner or family member.^8^ This amounts to 137 women, a day. In Australia, in the year 2021/2022, family violence victims accounted for 32% of all hospitalisations related to assault.^9^ For women aged 15–44, family violence is the foremost preventable cause of death, disability and health complications.^10^ The economic ramifications are also substantial. The combined expenses for healthcare, administration and social welfare linked to family violence cost the Australian economy approximately $21.7 billion AUD annually.^11,12^

Hospitals play an instrumental role in offering support to those affected by family violence.^13,14^ Given the high community lifetime and point prevalence of family violence, many patients have either current or previous experiences of family violence.^1,15^ Victim-survivors may be more likely to access healthcare services (such as public hospitals) than other professional services.^13^ Pathways to care may not be straight-forward. Many victims of physical and sexual violence to do not access care, when they need it, and/or may be unable to attend specialist services due to technology-assisted monitoring of their locations by the person using violence against them.^16,17^

Recognising the signs and appropriately addressing family violence is a crucial competence for all hospital professionals, regardless of the boundaries of specific disciplines such as social work, nursing or medicine.^18,19^ Individuals affected by family violence may seek care across various hospital departments, from emergency units to scheduled admissions or outpatient clinics, and may disclose to professionals of any discipline.^15^ The effectiveness of universal family violence screening has been debated.^20,21^ However, the need for routine inquiry within healthcare settings that have established training and protocols is commonly recommended, especially when indicators of family violence are present.^18,20,22–24^ Feedback from women with lived-experience of abuse emphasises the importance of persistent inquiry about potential family violence, even if initial conversations do not lead to disclosures, as it can take time for victim-survivors to develop trust in a service.^25^ Hence, training in family violence identification and intervention is essential for all hospital staff, ensuring they are well placed to conduct screening and provide a supportive response at any stage of a patient’s healthcare journey.^18,24^

Many healthcare workers are not cognisant of this pivotal role and express feelings of inadequacy and unpreparedness recognising and assisting patients with family violence issues. Research across various nations and healthcare settings reflects a consistently low clinician knowledge base when it comes to family violence inquiry.^26–31^ A study capturing the perspectives of Victorian-based health professionals in Australia revealed that only 20% felt equipped to handle family violence cases.^30^ Failures in preparedness to respond to violence have significant clinical ramifications. A study from South Australia indicated that of victim-survivors treated at an emergency department, just 8.5% self-presented describing family violence as the reason for their presentation, despite 44.6% having assault related injuries.^32^ Such findings highlight the need for robust service provision strategies, encompassing both structural procedures and high-quality training of clinical staff in family violence screening and response.

What is less clear is the type, duration and intensity of training that is required, to make meaningful and sustainable changes to health clinicians’ knowledge, confidence and skill levels in this area. At a cohort level, previous research has shown that a minimum of 7–9 h training in family violence is needed for at least 50% of hospital clinicians to rate themselves as having moderate levels of knowledge.^30,33^ Improving clinicians’ confidence in their skills required even more investment, with 10–15 h training required to reach moderate confidence levels in hospital clinicians. Family violence training workshops of 1–2 days duration have been shown to have some effect at improving self-rated knowledge, attitudes and service culture in clinicians in the United States and Britain.^34–36^ Some improvement in practice was also reported in these studies, including enhanced enquiry skills.^36^ However, others reported that training did not increase the rates of identification of patient domestic violence experiences, and researchers noted that potential and actual harmed still occurred in the units, after the training had been implemented (including breaches of confidentiality and a mother and infant being discharged to return to a home with an abusive partner).^35,36^

Recent previous Australian research evaluated a clinical champions model for training healthcare workers to better respond to family violence. Clinical champions have been utilised in healthcare to advance knowledge and skills in many areas including hand-hygiene and dementia care.^37^ Within the framework of a champions model, which included a community of practice, a ‘Family Safety Advocate’ (FSA) programme was developed at Royal Melbourne Hospital and evaluated across both allied health and nursing clinician cohorts.^38,39^ The training model involved a minimum of 9 h training and was effective at improving clinicians’ knowledge, confidence and specific family violence clinical skills (indicators, enquiry, responding). These gains were largely maintained over 12- to 15-month follow-up periods (supported by an ongoing community of practice) in allied health clinicians.^38^ In the nursing cohort, however, knowledge maintenance was less strong at follow-up (although still higher than baseline levels), and a higher proportion of participants were lost to follow-up and did not participate in the community of practice.^39^

In Victoria, Australia, the Strengthening Hospital Responses to Family Violence (SHRFV) initiative was launched by the state government to implement recommendations from a Royal Commission into Family Violence.^19,40^ This initiative aimed to improve public health services’ responses to family violence by providing grant funding for whole-of-hospital transformational change projects in all state health services. The current study sought to evaluate the impact of the whole-of-hospital transformational change project in a large, tertiary, trauma metropolitan hospital, participating in the SHRFV initiative.^41^ Recent comparison data indicate that the initiative was effective over 3 years at improving staff family violence knowledge in a rage of areas, relative to baseline at a whole of hospital clinician-cohort level.^42^ The current study sought to evaluate the impact of training intensity on clinician self-reported knowledge, confidence and clinical skills in responding to disclosures of family violence. Understanding the impact of broad-scale transformational change projects is important for establishing whether the resources required to embed these programmes result in meaningful improvements in staff skills, and whether this confers improvements in patient care, safety and well-being. It is also important to determine the extent of training that is required to improve clinician knowledge and confidence working in this area, and to establish whether training results in changes in clinical practice. The study was approved by Melbourne Health Human Research Ethics Committee (project number: HREC/17/MH/283), conducted in conjunction with the Helsinki declaration, with all hospital research staff having Good Clinical Practice certificates.

## Methods

A cross-sectional survey, post training implementation study design was utilised. The Checklist for Reporting of Survey Studies (CROSS) is provided in Supplemental Appendix 1.^43^ Data were collected for the study over a 6-week period in November and December 2020.

### Participants and setting

Participants were sourced from Royal Melbourne Hospital, a large government tertiary, tier-1, trauma hospital in Melbourne, Australia. Consistent with other state hospitals, the hospital was participating in the SHRFV initiative at the time of the survey. The hospital features several divisions spanning both acute and subacute settings, including emergency medicine, trauma and community rehabilitation. The survey was open to all hospital employees with a clinical role. Provided participants met the inclusion criteria, there we no further exclusion criteria. All clinical staff with known work email addresses (medical = 660, nursing = 1,829, allied health = 549) were invited to participate online via an email invitation.

### Training intervention

Staff training at the Royal Melbourne Hospital sat within a broader transformational change project as documented previously.^41^ Commencing in 2018, a hospital-wide family violence management and response protocol was implemented. In brief, this broad reaching protocol included the establishment of a specialised Family Safety Team (FST), linkages with existing digital infrastructure to support clinicians responding to patient family violence experiences, creation of a family violence working group and establishment of a programme of research.

The evidence-based training intervention aligned with research and best practices^44,45^ and was rolled out by the internal FST. Training ranged from brief sessions (e.g. 30–60 min), moderate length sessions (1–6 h), to an extensive Clinical Champions programme (9 h +) with community practice involvement, and are summarised below.

#### Short-duration training

Clinicians across the hospital attended discrete, time-limited training sessions online (conducted live in real time) or in-person which included:

- Module 1: Sensitive practice introduction (30–60 min). Rates of family violence, recognising signs in patients, why assisting is important.
- Module 2: Sensitive practice application. How to ask patients about family violence, how to respond and how to support (30–60 min).
- Refresher: Recap of modules 1 and 2 (60 min).
- Discipline-specific family violence clinical response training (30–120 min).
- Managerial training to support staff who may be experiencing family violence (60 min).

#### Family safety advocate training (clinical champions)

This 9-h programme offered comprehensive training on working with patients experiencing family violence. Throughout 2018–2019, the first segment (3 h) was presented live, in-person at the health service and covered introduction to family violence, healthcare responses, hospital protocols, research insights and determining and evaluating risk. The next live, in-person segment (6.5 h) centred on the Common Risk Assessment Framework Level 3 (CRAF3) and was presented by external facilitators (who attended onsite at the health service).^46^ The CRAF3 training focused on knowledge of, and skill development using, the family violence risk assessment framework in operation in the state at the time of the training. Topics included risk assessment, safety planning and networking. In 2020, due to the COVID-19 pandemic, the FSA training changed to be comprised of a 1-h online self-paced learning module, followed by an 8-h (full day) live interactive online seminar. The online seminar covered all of the topics listed above in the 2018–2019 training, but was provided in-house by [Hospital Name] trainers, rather than by an external agency for the CRAF3 portion. Post-training support involved monthly clinical supervision, quarterly meetings and access to a focussed workshop on resisting collusion with people using family violence (6 h). This additional training spanned 15-months post-FSA training.

### Survey tool

The Assisting Patient/Clients Experiencing Family Violence: Royal Melbourne Hospital Clinician Survey tool was utilised to assess clinician self-reported family violence response knowledge and confidence, in a cross-sectional, single-stage manner.^33^ The survey tool was administered via an online platform, and responses were collected anonymously. The survey was developed for use in Victorian healthcare settings, has been utilised in a number of previous studies, totalling *N* = 662 participants, and shows good internal reliability (Cronbach’s alpha of 0.83).^31,33,47,48^ In addition to demographic questions, there are seven key questions pertaining to family violence knowledge and skills, with Likert-type or ordinal response options. The tool also has qualitative self-report text-box data sections regarding specific knowledge of key family violence clinical skills (see Supplemental Appendix 1). The tool has also been shown to discriminate within subject changes in family violence knowledge occurring following training, in two longitudinal studies.^39,49^ This article presents the quantitative survey results with qualitative results presented elsewhere (submitted). Survey data were stored on a secure computer network and only accessible by the research team. As approved by the Research Ethics Committee, consent was implied on submission of survey answers and participants were unable to withdraw following submission of responses.

### Statistical analysis

Based on knowledge of participation rates in a previous similar survey implemented at the hospital 3 years prior (534 participants, 17% response rate),^33^ it was estimated that a similar response rate would be obtained. Sample size calculations indicated that a response rate of 342 participants (from the 3,038 available staff email addresses) was required to obtain results representative of the target population (95% confidence interval and 5% margin of error). The survey was open to all clinical staff at the hospital, with the only limiting factors being access to work emails and willingness to participate. All data were analysed using SPSS (version 26, IBM). A two-sided alpha value of less than 0.05 was considered statistically significant for all analyses. Differences in self-reported rates of knowledge, confidence, screening and frequency of working with clients who disclose family violence, between professional groups were analysed using Kruskal–Wallis. Post hoc analysis using pairwise comparison was undertaken to explore significant effects, with Bonferroni correction applied. Effect sizes were calculated using Cohen’s *f* coefficients with magnitude interpreted as 0.1 = small, 0.3 = medium and 0.5 = large.^50^ This non-parametric analysis was chosen due to the ordinal nature of data. Nominal data relating to demographics, understanding of indicators, ability to inquire about violence and how to manage disclosures and were compared using chi-squared analyses. Post hoc analysis using Bonferroni correction was applied.

## Results

### Participants

Five hundred and twenty-six staff participated in the online survey (see Table 1 for demographics). Staff from a wide range profession groups and clinical areas participated, with an overall response rate of 17.10%. Differences in participation rate across professional group were identified (Allied Health 44.63%, Nursing 11.65%, Medical 10.30%). Participants represented a fairly experienced cohort, with more than 65% having at least 6-years experience in their profession, and 45% with 10 years or more. More than three-quarters of respondents identified as female gender. From the total cohort, 29.66% (*n* = 156) had undertaken no training in family violence, 53.04% (*n* = 276) had undertaken some family violence training and 17.87% (*n* = 94) had trained in the hospital’s clinical champions FSA programme. No medical staff had trained as advocates. Training groups were defined by self-report. Participants who indicated they had not undertaken training and endorsed zero minutes of training time were categorised as ‘No Training’, those who indicated that they had trained in the ‘FSA’ (clinical champions) programme were included in this category. All remaining respondents were categorised as ‘Some Training’. Between the two groups who had participated in training, the FSAs had undertaken, on average, 10 h more training in family violence than those in the Some Training cohort. However, there was a large range in the number of hours of training across both groups.

**Table 1.** Demographic characteristics of participants according to training intensity level.

Participant category	Total sample	No training	Some training	FSA training
*N*	526	156	276	94
Profession/subgroup, *N* (% of total)				
Nursing	213 (40.49)	86 (55.13)	102 (36.96)	25 (26.60)
Acute	137 (26.05)	63 (40.38)	58 (21.01)	16 (17.02)
Emergency department	44 (8.37)	14 (8.97)	25 (9.06)	5 (5.32)
Subacute	22 (4.18)	6 (3.85)	13 (9.05)	3 (3.19)
Other	10 (1.90)	3 (1.92)	6 (2.17)	1 (1.06)
Allied Health	245 (46.58)	33 (21.15)	143 (51.81)	69 (73.40)
Physiotherapy	53 (10.08)	9 (5.77)	40 (14.49)	4 (4.26)
Social work	41 (7.79)	0 (0)	0 (0)	41 (43.62)
Occupational therapy	40 (7.60)	3 (1.92)	31 (11.23)	6 (6.38)
Clinical nutrition/dietetics	18 (3.42)	3 (1.92)	12 (4.35)	3 (3.19)
Speech pathology/audiology	20 (3.80)	4 (2.56)	15 (5.43)	1 (1.06)
Psychology	17 (3.23)	0 (0)	8 (2.90)	9 (9.57)
Other	56 (10.65)	14 (8.97)	37 (13.41)	5 (5.32)
Medical	68 (12.93)	37 (23.72)	31 (11.23)	0 (0)
Acute	32 (6.08)	15 (9.62)	17 (6.16)	0 (0)
Emergency department	10 (1.90)	8 (5.13)	2 (0.72)	0 (0)
Subacute	8 (1.52)	3 (1.92)	5 (1.81)	0 (0)
Outpatients	8 (1.52)	5 (3.21)	3 (1.09)	0 (0)
Rehabilitation	4 (0.76)	3 (1.92)	1 (0.36)	0 (0)
Other	6 (1.14)	3 (1.92)	3 (1.09)	0 (0)
Years of experience in profession, *N* (%)				
<1 year	29 (5.51)	17 (10.90)	10 (3.62)	2 (2.13)
1–5 years	136 (25.86)	24 (15.38)	84 (30.43)	28 (29.79)
6–10 years	122 (23.19)	38 (24.36)	69 (25)	15 (15.96)
>10 years	239 (45.44)	77 (49.36)	113 (40.94)	49 (52.12)
Age bracket				
<25	23 (4.37)	7 (4.49)	13 (4.71)	3 (3.19)
25–29	101 (19.20)	19 (12.18)	63 (22.82)	19 (20.21)
30–39	186 (35.36)	61 (39.10)	97 (35.14)	28 (29.79)
40–49	122 (23.19)	38 (24.36)	54 (19.57)	30 (31.91)
50–59	65 (12.36)	19 (12.18)	37 (13.41)	9 (9.57)
60–64	23 (4.37)	10 (6.41)	11 (3.99)	2 (2.13)
65+	6 (1.14)	2 (1.28)	1 (0.36)	3 (3.19)
Gender identity, %				
Female	414 (78.71)	111 (71.15)	217 (78.62)	86 (91.49)
Male	102 (19.39)	42 (26.92)	56 (20.29)	4 (4.26)
Non-binary	3 (0.57)	0 (0)	0 (0)	3 (3.19)
Transgender	0 (0)	0 (0)	0 (0)	0 (0)
Different identity	0 (0)	0 (0)	0 (0)	0 (0)
Prefer not to say	7 (1.33)	3 (1.92)	3 (1.09)	1 (1.06)
Prior family violence training				
Mean hours	4.31	0	3.25	14.60
Standard deviation hours	7.37	0	5.23	9.14
Range hours	0–60	0	1–60	4–60

FSA: Family Safety Advocate.

Allied Health staff were moderately overrepresented in the sample, and medical staff moderately under-represented, relative to the composition of clinical cohorts within the hospital. The FSAs were predominantly from the Allied Health professions. All computer log-ins provided a unique (anonymous) respondent ID. No ID duplications were present in the dataset indicating that the likelihood of multiple participations by the same staff member was low.

### Knowledge, confidence and screening

To explore differences in self-reported knowledge, confidence, screening rates and frequency of working with those who disclosed experience family violence, a series of Kruskal–Wallis analyses were conducted (see Table 2 for associated mean ranks). There was a large, statistically significant difference between self-reported family violence knowledge based on the amount of family-violence training received, *H*(2) = 208.52, *p* *<* 0.001, *f* = 0.81. Post hoc analysis using Bonferroni correction indicated that clinicians with No Training reported significantly lower levels of knowledge than clinicians with Some Training (*p* < 0.001) and clinicians who had completed the FSA (Clinical Champions) training (*p* < 0.001). Additionally, FSAs reported significantly greater levels of knowledge than those with Some Training (*p* < 0.001).

**Table 2.** Mean ranks for self-reported knowledge, confidence, screening rates and frequency of working with clients who disclose family violence based on amount of training received.

Participant self-rated area	No training	Some training	FSA training
Knowledge	150.1	276.1	414.6
Confidence	162.5	270.4	410.8
Frequency of screening	194.2	267.6	366.4
Frequency of working with disclosing clients	226.5	255.0	350.0

FSA: Family Safety Advocate.

A similar pattern of results was seen for clinician confidence addressing family violence issues experienced by patients. There was a large, statistically significant difference between self-reported confidence across training levels *H*(2) = 171.83, *p* < 0.001, *f* = 0.70. Pairwise comparison with Bonferroni correction again revealed having Some Training resulted in significantly greater confidence than having No Training. Those with FSA training also reported significantly greater confidence compared to those with Some Training and No Training (*p* < 0.001 for all comparisons).

A moderate, statistically significant difference was seen in self-reported frequency of screening patients for family violence based on training level, *H*(2) = 80.37, *p* < 0.001, *f* = 0.43. Those with No Training reported screening patients significantly less often than those with Some Training. FSAs reported significantly greater frequency of screening clients than those in both other training groups (*p* < 0.001 for all comparisons).

Finally, for frequency of working with clients who disclose family violence, Kruskal Wallis analysis indicated a significant difference based on amount of training, *H*(2) = 45.77, *p* < 0.001, *f* = 0.31. Pairwise comparisons indicated no significant difference between clinicians with No Training and Some Training. FSAs, however, reported significantly greater frequency of working with disclosing clients relative to both clinicians with Some and No Training (*p* < 0.001).

### Clinical skills

**Figure 1.**
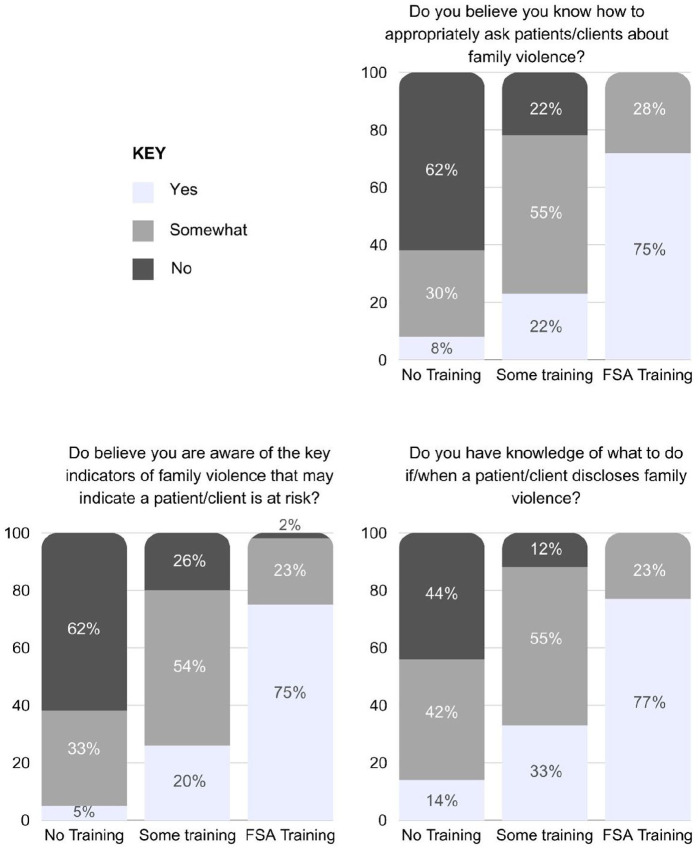
Specific family violence clinical skills by response and training level.

A series of chi-squared analyses with Bonferroni correction indicated differences in specific family violence clinical skills for identifying indicators of violence, asking about violence and managing disclosures. These results are presented in Table 3, and percentage responses by question and training level in Figure 1. For all questions pertaining to specific family violence clinical skills, those with No Training endorsed having less knowledge than those with Some Training, and FSAs. Specifically, clinicians with No Training were significantly less likely than both other groups to respond *Yes*, or *Somewhat* for knowing how to ask about family violence. In contrast FSAs were significantly more likely to response definitely *Yes*, or *Somewhat* to this question. This was also seen for endorsed knowledge of indicators of family violence. Clinicians with No or Some Training were significantly less likely to respond *Yes* and were more likely to respond *No*. The opposite pattern was seen among FSAs. When asked about knowledge of how to respond to disclosures, those with No Training were the least likely to respond *Yes* or *Somewhat*, whilst those with FSA training were the most likely to respond *Yes* or *Somewhat* (*p* < 0.001 for all comparisons).

**Table 3.** Summary of self-reported clinical skills across training groups.

Clinical skill area	No training, *n* (%)	Some training, *n* (%)	FSA training, *n* (%)	χ^2^ test of independence (*p*-value)	Comparisons that reached statistical significance and direction (higher >lower)
Indicators of FV				212.19 (<0.001)	FSA training>some training>no training
Yes	7 (4.5)	73 (26.4)	70 (74.5)		
No	97 (62.2)	54 (19.6)	2 (2.1)		
Somewhat	52 (33.3)	149 (54.0)	22 (23.4)		
How to ask about FV				201.15 (<0.001)	FSA training>some training>no training
Yes	13 (8.3)	63 (22.8)	68 (72.3)		
No	97 (62.2)	61 (22.1)	0 (0)		
Somewhat	46 (29.5)	152 (55.1)	26 (27.7)		
How to manage disclosures				160.58 (<0.001)	FSA training>some training>no training
Yes	22 (14.1)	90 (32.6)	72 (76.6)		
No	69 (44.2)	32 (11.6)	0 (0)		
Somewhat	65 (41.7	154 (55.8)	22 (23.4)		

FSA: Family Safety Advocate.

## Discussion

The results of the current study support the effectiveness of intensive clinical training scaffolded by a community of practice in enhancing knowledge and preparedness to respond to family violence in hospital clinicians. In this large cohort of clinical staff from across disciplines, those with no training in family violence consistently rated their knowledge and confidence working in this area lower than staff who had received training. Not surprisingly, staff who had been trained as clinical champions (FSAs) reported consistently higher knowledge, confidence and skills awareness, than staff with no training and those with shorter duration training.

At a mean rank level, staff with some training rated their knowledge and confidence levels at least two-thirds higher than those with no training (mean training hours 3.25, SD 5.23). Those trained as clinical champions (mean training hours 14.60, SD 9.14), rated their knowledge and confidence at least 50% higher than staff with some training. A similar pattern of results, with more pronounced differences, was seen for specific family violence clinical response skills. Rates of those responding definitively *Yes* to having knowledge of the indicators of family violence increased dramatically with training exposure (No training – 5%, Some training – 26%, FSAs – 74%), as did knowledge of how to ask patients about family violence (No training – 8%, Some training – 23%, FSAs – 75%) and how to manage disclosures (No training – 14%, Some training – 33%, FSAs – 77%). Thus, at the level of key family violence skills, some training is beneficial over no training; however, the extra resourcing required to train clinical champions (9+ hours of teaching), conveys a significant level of benefit over short-duration training. These results lend further support for the utility of family violence clinical champions training models, building on the existing results in two previous studies at the same health service.^38,39^ Numeric findings of this article also converge with qualitative outcomes, which reflect a greater depth of understanding among clinicians with more intensive training (Troy, Rushan, Withiel, Felmingham & Fisher, submitted).

Although the stronger self-reported knowledge and confidence levels in the FSA trained group may largely reflect their higher number of family violence training hours, it is also likely that having a community of practice to participate in, to consolidate and maintain knowledge and skills, was of benefit. Confidence can take longer to build, than self-reported knowledge, as clinicians require time to utilise and consolidate newly learned skills. Evidence to support this can be seen in baseline study data at the hospital, indicating that a higher number of training hours were required for clinician ratings of confidence working in the area of family violence to match their self-rated knowledge levels.^33^ This was also borne out in evaluation of the initial allied health training cohort of clinical champions (FSAs), where confidence ratings lagged knowledge improvement ratings immediately post training, but rose to comparable levels over 9- and 12–15-month follow-up periods.^38^

The results of this study also suggest, at least at a self-report level, that training in family violence changes clinical practice. Clinicians with some training self-reported screening clients more often than those without training, and the FSAs clinical champions group reported screening levels that were significantly higher again, relative to staff with some training. This was further substantiated by the FSAs endorsing a much higher frequency of working with patients who had disclosed family violence. There is further objective evidence within the health service to support an increase in screening rates. At the commencement of the initiative there was no method for quantifying, tracking or recording family violence screening and no standardised way in which this was conducted. With the implementation of the hospitals new electronic medical record (rolled out progressively between mid-2019 and late 2020), a standardised work-flow for family violence screening was embedded and saw a steady upswing in utilisation. The earliest available data (4-month period, November 2020 to February 2021) indicates usage rates of 3,507 times a month on average during healthcare encounters. At the time of writing of this article, the most recently available internal audit report indicated that the workflow was utilised an average of 4,972 times per month (November 2023 to February 2024), with consistent increases in the intervening years. Family violence training has continued throughout the service since the survey data was obtained. This is likely to have contributed to the increase in screening tool usage.

There are limitations to the current study. The response rate for the survey was not optimal, and differed across clinician profession groups. As such, the sample may not be representative of all clinicians at the service. Further to this point, allied health staff were over-represented in the sample, and medical staff underrepresented, relative to their cohort sizes in the hospital. There are likely to be several reasons for this, firstly the research team were located within Allied Health, giving the clinicians in this service a higher exposure to both the training initiative and information about the survey project. Medical staff also had a consistent pattern of less engagement with the family violence research stream.^33,48^ Finally, the research was only conducted at a single hospital and the results many not be generalisable to others within the state, funded under the same SHRFV initiative.

This suite of research is the first to comprehensively evaluate the impact of the SHRFV project in a Victorian public hospital, including both baseline research studies, and analysis of clinician skills levels, overtime and by training level.^15,33,41,42,48,49,51^ To our knowledge, the service is also the first to trial and evaluate the effectiveness of healthcare clinical champions model in the area of family violence clinical response. The results dovetail with the preliminary results tabled in the SHRFV SAFE Audit Tool report which spanned a similar data collection time period.^52^ Data collection for this report was undertaken as a snapshot audit at a single time point, in 18 of the 88 participating services Victorian health services. It indicated that many services had made moderate to strong gains in *foundational* aspects of the SHRFV implementation model (policies, guidelines, culture, collaboration and integration, governance and leadership), moderate gains in the area of *capacity* (staff support, education and training) and *practice* (patient identification and response) and moderate-to-low gains in the area of *investment* (infrastructure quality improvement, diversity). The report recommendations called for greater investment and for government commitment to funding of permanent family violence-specific roles to sit within health services to ensure early gains are sustained.^52^ This aligns with the findings of the current research, that indicates in-depth training is the most efficacious at improving clinician, knowledge, confidence and practice and that beyond training, a community of practice is important for consolidating skills. Both training and maintaining skills require resourcing, and without a maintenance of resourcing, it is less likely that a high level of skills in family violence clinical response will be sustained, over time.

## Conclusion

This broader suite of research indicates that healthcare services, coming from a low baseline skill level, can enact meaningful change in clinician preparedness to respond to family violence over a relatively short (3-year) time frame. Engagement with leadership, supporting policies and procedures and a dedicated well-resourced team, are key to implementing such changes. The work commenced through the SHRFV initiative in Victoria made initial steps to implement the healthcare reforms recommended by the Victorian Royal Commission into Family Violence. It has also enabled many healthcare services in the state to begin to align with international best-practice guidelines for healthcare family violence clinical response. However, from the 2024/2025 fiscal year, the initiative will no longer receive any funding from within the state government healthcare budget. It remains to be seen whether the early gains made within the states healthcare sector can be maintained over time, particularly at healthcare services where internal funding is not secured.

The pivotal role healthcare services play as a gateway function support to people experiencing family violence to access assistance remains a paramount part of a well-functioning community response to this issue. Adequately training and resourcing healthcare professionals to do this work effectively, and safely, is a key component. Lasting culture change that recognises the importance of this work in health, and a commitment from both leaders and funders to support this work, are key to enacting lasting and meaningful improvements.

## Supplemental Material

sj-docx-1-whe-10.1177_17455057241286552 – Supplemental material for How much training is enough? Evaluating clinician self-reported family violence response skills following a 3-year transformational change project in a major trauma hospital Supplemental material, sj-docx-1-whe-10.1177_17455057241286552 for How much training is enough? Evaluating clinician self-reported family violence response skills following a 3-year transformational change project in a major trauma hospital by Caroline A Fisher, Catherine Rushan and Toni D Withiel in Women’s Health

sj-docx-2-whe-10.1177_17455057241286552 – Supplemental material for How much training is enough? Evaluating clinician self-reported family violence response skills following a 3-year transformational change project in a major trauma hospital Supplemental material, sj-docx-2-whe-10.1177_17455057241286552 for How much training is enough? Evaluating clinician self-reported family violence response skills following a 3-year transformational change project in a major trauma hospital by Caroline A Fisher, Catherine Rushan and Toni D Withiel in Women’s Health

## Declarations

**Ethics approval and consent to participate:** This study was approved by the Melbourne Health Human Research Ethics Committee, HREC Reference Number: HREC/17/MH/283, SSA Reference Number: SSA/17/MH/390, Local Project Number: 2017.282. As approved by the Research Ethics Committee, consent was implied on submission of survey answers, participation was anonymous and participants were unable to withdraw following submission of responses.

**Consent for publication:** Prior to participating in the survey, potential participants were informed of the following, in writing: ‘The results of this survey will be used to develop appropriate and relevant training modules for clinicians in the area of assisting patients/clients experiencing family violence. The survey results will be disseminated to RMH (Royal Melbourne Hospital) and [partner service] via informational email, and may be submitted for publication’. As approved by the Research Ethics Committee, consent was implied on submission of survey answers. and participants were unable to withdraw following submission of responses.

**Availability of data and materials:** As per ethics approval, all data are retained by the authors. Enquiries regarding the data can be made via the corresponding author.
