# Supplementary material for: How much training is enough? Evaluating clinician self-reported family violence response skills following a 3-year transformational change project in a major trauma hospital
Source: Womens Health (Lond). 2024 Oct 18;20:17455057241286552. doi: 10.1177/17455057241286552 (PMC11489942; doi:10.1177/17455057241286552)
Supplement: sj-docx-1-whe-10.1177_17455057241286552 – Supplemental material for How much training is enough? Evaluating clinician self-reported family violence response skills following a 3-year transformational change project in a major trauma hospital [file sj-docx-1-whe-10.1177_17455057241286552.docx]

Appendix 1

Assisting Patient/Clients Experiencing Family Violence: Royal Melbourne Hospital Clinician Survey

The *Family Violence Protection Act 2008* defines family violence as including a range of behaviours such as physical and sexual abuse; emotional or psychological abuse; economic abuse; behaviour that is threatening, or coercive, or in any way controls or dominates that person or causes them to feel fear. This definition also includes behaviours that cause a child to witness or hear or otherwise be exposed to the effects of family violence. Family violence also includes abuse to elderly persons which can include physical, sexual, financial, psychological, social and/or neglect. We are seeking information about the knowledge of [Health Service 1] clinicians in working with *patients/clients* experiencing family violence. This information will help guide training and development initiatives in this area.

Please complete the following brief survey.

1. What is your profession?

*Drop down box with all Allied Health Professions, nursing (by area) and doctors (by area).*

1. How long have you worked in your profession?

□ <1 year □ 1-5 years □ 6-10 years □ >10 years

1. Which of the following best describes your gender identity?

□ Female □ Male □ Non binary/Gender fluid

□ Different Identity □ Prefer not to say

1. What is your age group in years?

□ Under 25 □ 25-29 □ 30-39 □ 40-49 □ 50-59

□ 60-64 □ 65+

5) Are you an RMH Family Safety Advocate?

□ Yes □ No

1. Have you undertaken any training in Family Violence? Check all boxes that apply

*List with all available Family Violence training at service*

1. How many hours of family violence training have you received?

Please tick one box on each line:

|  | None | 1-3hrs | 4-6hrs | 7-9hrs | 10-15hrs | 16+hrs |
| --- | --- | --- | --- | --- | --- | --- |
| Self-taught |  |  |  |  |  |  |
| In-service session/s at RMH |  |  |  |  |  |  |
| In-service session/s at another hospital |  |  |  |  |  |  |
| One-off workshop, external to hospital training |  |  |  |  |  |  |
| External short course |  |  |  |  |  |  |
| During your professional training |  |  |  |  |  |  |
| Other (with brief description):  __________________________ |  |  |  |  |  |  |

If you have received family violence training, has any of this training occurred in the last 2 years?

□ Yes □ No

1. How would you rate your knowledge of working clinically in the area of family violence?

□ No knowledge □ Limited knowledge □ Moderate knowledge □ Strong knowledge □ Very knowledgeable

1. How confident are you working clinically in the area of family violence?

□ Not at all confident □ A little amount confident □ Moderately confident □ Confident □ Very confident

1. How often do you screen patients/clients for their experiences of family violence?

□ Never screen □ Rarely screen □ Sometimes screen □ Often screen □ Always screen every client

1. How often do you work with patients/clients who have disclosed family violence, to your knowledge?

□ Never □ Very Seldom □ Sometimes □ Often □ Most of the time □ Always

1. Do you believe you know how to appropriately ask patients/clients about family violence?

□ Yes □ Somewhat □ No

If you answered *Yes*, or *Somewhat*, please describe how you would do this:

1. Do believe you are aware of the key indicators of family violence that may indicate a patient/client is at risk?

□ Yes □ Somewhat □ No

If you answered *Yes*, or *Somewhat*, please describe what these are:

1. Do you have knowledge of what to do if/when a patient/client discloses family violence?

□ Yes □ Somewhat □ No

If you answered *Yes*, or *Somewhat*, please describe what you do/would do:

1. Please indicate if any of the factors below are challenges for you in addressing family violence?

(You can tick more than one box)

□ Time limitations when seeing a patient/client

□ I don’t know what to do or say

□ Patient/client’s reluctance to disclose when asked

□ Concern about offending the patient/client or affecting rapport

□ The patient/client’s partner/child/parent (i.e. suspected perpetrator) is present

□ Another vulnerable person is present (i.e. children)

□ Concerns about staff safety in asking questions about family violence and initiating action

□ Lack of supporting policies and procedures

□ The topic of family violence is uncomfortable

□ Language barriers

□ Privacy issues in the clinical area in which I work

□ Little or no access to supervision that supports safe and reflective practice in this area

□ I have no difficulties in addressing family violence

□ Other, please specify:

Thank you for taking the time to complete this survey.

If completing this survey has raised issues for you, or caused you to feel distressed, please note that [Health Service] Employees can access the Assistance Program, EAP (number and details provided)

You can also contact other services, such as: (contact details for six local community organisations provided).
